# Editorial: Psychology and Neuropsychology of Perception, Action, and Cognition

**DOI:** 10.3389/fnhum.2022.875947

**Published:** 2022-03-18

**Authors:** Jane E. Aspell, Els Ortibus, Silvio Ionta

**Affiliations:** ^1^School of Psychology and Sport Science, Anglia Ruskin University, Cambridge, United Kingdom; ^2^Locomotor and Neurological Disorders, Department of Development and Regeneration, KU Leuven, Leuven, Belgium; ^3^Sensory-Motor Lab (SeMoLa), Department of Ophthalmology-University of Lausanne, Jules Gonin Eye Hospital-Fondation Asile des Aveugles, Lausanne, Switzerland

**Keywords:** brain, vision, movement, multisensory, sensorimotor

Translating multidisciplinary scientific knowledge into unified psycho-educational practices can improve the restoration and establishment of basic functions, such as using tools or interacting with others. For instance, low vision has a tremendous impact on writing, navigating, or playing in groups, but current interventions rely on specialized knowledge from different areas typically operating in isolation.

Following from the symposium “NeuroPedagogy of Vision and Beyond”, held at the Fondation Asile des Aveugles in May 2021, with the support of the Center Pédagogique pour élèves Handicapés de la Vue (CPHV) and the Frontiers Publishing Group, the present Research Topic collects 14 articles (11 original studies, three reviews) by world-leading neuroscientists, pedagogues, neuropsychologists, clinicians, and developmental psychologists. The articles describe state-of-the-art behavioral, psychophysical, and brain imaging studies of sensory-motor-cognitive loops in health and disease, within three main topics: perception, action, cognition (Table 1).

**Table 1:** Classification of included articles as a function of the RT's topics (P, Perception; C, Cognition; A, Action).

**#**	**Type**	**1st Author**	**Frontiers in**	**Class**	**Technique**	**Age**
1	Exp	Yang	Psychology	P	Behaviour	2–5 months
2	Review	Solovieva	Psychology	C	Behaviour	1 year
3	Exp	Ling	Psychology	C	Behaviour	3 years
4	Exp	Zacharov	Psychology	C	Behaviour	3–6 years
5	Exp	Alghamdi	HumNeur	P/C	Behaviour	5–7 years
6	Exp	Ye	Psychology	A/C	Behaviour	1–3 years
7	Exp	Farran	HumNeur	A/C	Behaviour	5–11 years
8	Exp	Fitamen	Psychology	A/C	Behaviour	5 years
9	Exp	Weibley	BehavNeur	A/C	fNIRS, behaviour, cognitive	8–14 months
10	Exp	Guan	Psychology	A/P	EEG, behavior	9–10 years; adults
11	Exp	Esposito	HumNeur	A/P	Behaviour	adults
12	Exp	Micheletti	HumNeur	A/P	Behaviour	5–12 years
13	Review	Ionta	HumNeur	A/P	Brain, behaviour	0–65 years
14	Review	Farah	Psychology	A/P/C	Brain, behaviour	0–65 years

Within the “*perception*” axis, Yang et al. provide new evidence about the development of binocular suppression mechanisms. The authors used a continuous flash suppression task to induce a conflict between the visual input (one perceptually dominant and one perceptually non-dominant image) delivered to one and the other eye, in 2–5-month-old infants. Only younger infants showed to perceive the non-dominant image, indicating that about 3 months the binocular suppression mechanisms are not fully formed yet.

Within the “*cognition*” axis, Solovieva and Quintanar highlight the influence of cultural factors on the so-called first year developmental crisis, involving radical psychological changes constituting the basis of the following motor, cognitive, social developments (Solovieva and Quintanar). This paper provides important information for optimizing child-adult interactions while building a reliable psychological context for the child's subsequent development. In the same “cognitive” vein, Ling et al. show that the properties of the task used to assess conditional reasoning skills can importantly affect the performance and, therefore, the establishment of the minimum age for appropriate conditional reasoning. The authors used a modified version of the dimensional change card sort (DCCS), including color as a key feature of target objects. While the DCCS task is typically solved around the age of 5, this manipulation enabled 3-year-old children to succeed, highlighting the importance of accounting for possible implicit biases of commonly accepted procedures (Ling et al.). Using a similar experimental approach, Zacharov et al. illustrated the impact of autism on cognitive flexibility. The authors administered a DCCS task, in combination with a reverse categorization task and a non-verbal cognitive age assessment, in children with and without autism. Autism was associated with worse performance in the DCCS task and disrupted the correlation between mental age and performance in both tasks. However, when the two groups were matched by mental age, their performance in the two tasks was not significantly different, highlighting the importance of fine classifications and precise evaluations of the methods used to assess cognitive skills.

Within the “perception-cognition” axis, Alghamdi et al. investigated the relationship between global intelligence and visual processing by measuring the speed of visual inspection, visuo-verbal interactions, and visuomotor control in 5–7-year-old children. The latter two were found to correlate with non-verbal intelligence scores and years of education and as such, might be possible targets in educational programs.

Along the line of Zacharov et al., but within the “*action-cognition*” axis, a specific focus on autism was adopted also by Ye et al. to illustrate the characteristics of gesture production in 2–4 year-old children with autism. Children with autism showed less behavioral regulation, social interaction, and joint attention gestures. However, similar to Zacharov et al., correcting the gesture performance by the communication score changed the outcomes. Without correction, children with autism exhibited fewer gestures both with and without accompanying vocalization. With the correction, only the production of gesture without vocalization was lower in children with autism with respect to controls. In line with Ling et al. and Zacharov et al., the work by Ye et al. also underlines the importance of finely evaluating the assessment tools themselves, and not only the assessed populations. The relationship between cognitive and motor skills permeates three other articles. First, Farran et al. investigated the impact of physical disability on spatial cognition. They administered mental rotation, spatial programming, and virtual navigation to two groups of physically impaired children (differentiated by the need or not of wheelchair use) and controls. The performance of the two groups with physical disabilities was lower than controls, but did not vary between wheelchair users or not. This suggests that physical disability affects spatial cognition to a degree large enough that further differentiations as function of contextual factors (wheelchair) are minimal. Second, Fitamen and Camos show the benefits of motor activity on subsequent memory processes. These authors asked 5-year-old children to perform a memory task after a game-like and an exercise-like motor activity (in counterbalanced order) which both involved the objects used in the subsequent memory task. Children performed better in the exercise-like memory trials when they performed the exercise-like motor activity before the game like activity. Conversely, when they had the game-like motor activity before the exercise-like motor activity, there was no difference in their performance with exercise-like and game-like memory trials. This shows the superiority of game-like activities in establishing better-lasting memories. Third, focusing on the same age range and complementing behavioral observations of Solovieva and Quintanar, Weibley et al. described the cortical correlates of motor and cognitive skills. The authors monitored brain activity in the prefrontal cortex of children aged between 8 and 14 months while they were performing active and passive motor and attentional tasks. Within each category, active tasks were associated with higher prefrontal activity compared to passive tasks, highlighting the importance of active involvement in daily activities for motor-cognitive development (Weibley et al.).

Within the “*action-perception*” axis, Guan et al. studied the effects of motor activity on a subsequent perceptual task in 9–10-year-old children and adults (Guan et al.). Using EEG, they investigated the influence of a previous handwriting condition on the neural correlates of a subsequent visual word recognition task. They showed that in adults, but not in children, the brain activity during the visual perception task was lateralized toward the hemisphere dominant for language (left). The authors discuss their findings with respect to the importance of maintaining handwriting training in the digital era. A similar focus was adopted by the study by Esposito et al. that analyzed the consequences of early visual deprivation on the development of head-trunk coordination movements. The authors recorded movement kinematics from the head and trunk of young adults who were congenitally blind and controls performing a head-pointing task while voluntarily immobilizing (or moving) the trunk. While movement analysis showed a head-trunk coordination impairment in congenitally blind participants, their performance in the task was not significantly different from controls. This supports the plasticity of visuo-motor interactions, in that compensatory mechanisms can enable the achievement of goals through alternative strategies. In the same visuo-motor vein, Micheletti et al. examined the relationship between motor impairment and visual skills. In over 100 children with developmental coordination disorder (DCD) they compared the sensitivity to global motion and global static form stimuli to those of controls. Results showed that the performance of children with DCD in the global motion task was worse than controls, and that motor impairment was linearly correlated with global form sensitivity and presented a quadratic correlation with global motion sensitivity. The authors discuss these findings with reference to the differentiation between dorsal (motion) and ventral (form) stream functions, which seem differentially affected by motor impairment. Such a neuro-behavioral approach in the context of visuo-motor interactions is extended by two review papers. The first one summarizes over 300 papers about neuropsychological evidence on (i) the neural correlates of vision, (ii) anatomo-functional brain dynamics associated with the development of visual, motor, and visuo-motor skills in health and disease and across the life span (Ionta), and (iii) visuo-motor perspectives on relatively lower-level and more complex syndromes, such as strabismus, akinetopsia, DCD, and hemispatial neglect. The second one establishes a “*perception-action-cognition*” bridge, focusing on the neural and behavioral peculiarities of executive function in dyslexia (Farah et al.). Summarizing evidence from about 200 papers, this review highlights the importance of examining executive functions as possible early predictors of following reading/speaking deficits.

Overall, by strengthening the understanding of the neural bases of developmental disorders, the insights derived from the present Research Topic will hopefully provide a solid background to support interdisciplinary discussions among experts in sensory and/or motor disorders in the context of neuro-behavioral rehabilitation and training.

## Author Contributions

All authors listed have made a substantial, direct, and intellectual contribution to the work and approved it for publication.

## Conflict of Interest

The authors declare that the research was conducted in the absence of any commercial or financial relationships that could be construed as a potential conflict of interest.

## Publisher's Note

All claims expressed in this article are solely those of the authors and do not necessarily represent those of their affiliated organizations, or those of the publisher, the editors and the reviewers. Any product that may be evaluated in this article, or claim that may be made by its manufacturer, is not guaranteed or endorsed by the publisher.

